# Pro-inflammatory cytokines increase temporarily after adjuvant treatment for breast cancer in postmenopausal women: a longitudinal study

**DOI:** 10.1186/s13058-024-01898-3

**Published:** 2024-10-16

**Authors:** Agnes Lindholm, Marie-Louise Abrahamsen, Kristian Buch-Larsen, Djordje Marina, Michael Andersson, Jørn Wulff Helge, Peter Schwarz, Flemming Dela, Linn Gillberg

**Affiliations:** 1 Xlab, Department of Biomedical Sciences, University of Copenhagen, Blegdamsvej 3B, 2200 Copenhagen, Denmark; 2 Department of Endocrinology, Rigshospitalet, 2100 Copenhagen, Denmark; 3 Department of Oncology, Rigshospitalet, 2100 Copenhagen, Denmark; 4 Faculty of Health and Medical Sciences, University of Copenhagen, 2200 Copenhagen, Denmark; 5 Department of Biochemistry and Physiology, Riga Stradins University, Riga, Latvia

**Keywords:** Breast cancer, Cytokines, Inflammation, Metabolism, Chemotherapy, Side effects, Cardiometabolic

## Abstract

**Background:**

Breast cancer patients have an increased risk of cardiometabolic disease and for many patients, adjuvant therapy causes an altered lipid profile, insulin resistance and inflammation. Previous follow-up studies are inconclusive regarding the duration of therapy-induced inflammation. We examined the acute and persistent changes of adjuvant chemotherapy on inflammatory and metabolic health markers in breast cancer patients.

**Methods:**

Plasma levels of IL-6, IL-8, IL-10, IFN-γ, TNF-α, high-sensitivity C-reactive protein (hsCRP) and metabolic health parameters were analyzed before, shortly after and every six months up to two years after adjuvant chemotherapy treatment in 51 postmenopausal early breast cancer (EBC) patients, as well as in 41 healthy age- and BMI-matched controls. A target-specific multiplex assay was applied for cytokine measurements.

**Results:**

Before initiation of adjuvant therapy, plasma IL-8 levels were higher in EBC patients (31%, *p* = 0.0001). Also, a larger proportion of the patients had a hsCRP level above 2 mg/L (41%) compared to the controls (17%, Χ^2^ = 5.15, *p* = 0.023). Plasma levels of all five cytokines, but not hsCRP, were significantly increased after compared to before adjuvant chemotherapy (15–48% increase; all *p* ≤ 0.05). Already six months after ending chemotherapy treatment, all plasma cytokine levels were significantly reduced and close to pre-chemotherapy levels. Adjuvant chemotherapy caused a worsened lipid profile (increased triglycerides, lower HDL levels), insulin resistance and increased plasma insulin levels that remained high during the first year after chemotherapy.

**Conclusion:**

Postmenopausal women with EBC have temporarily increased plasma levels of pro-inflammatory cytokines after adjuvant chemotherapy. Although transient, the therapy-induced increase in plasma cytokine levels, together with dyslipidemia and insulin resistance, may contribute to cardiometabolic risk in breast cancer patients treated with adjuvant chemotherapy.

**Trial registration:**

The clinical trial (registration number NCT03784651) was registered on www.clinicaltrials.gov on 24 December 2018.

**Supplementary Information:**

The online version contains supplementary material available at 10.1186/s13058-024-01898-3.

## Background

In 2020 breast cancer became the most common type of cancer globally, accounting for 12% of new annual cancer cases [1]. Due to improved screening and treatment, the population of breast cancer survivors is growing. Indeed, the 5-year survival rate of early (stages I-III) breast cancer (EBC) is now greater than 90% [2]. This positive development in survival has brought attention to the undesirable short- and long-term consequences of adjuvant therapies (e.g., chemo- and radiotherapy). The short-term side effects of adjuvant chemotherapy are transient, such as nausea, fatigue, decreased appetite and hair loss [3]. Long-term side effects include weight gain, osteoporosis, fatigue, hypertension and metabolic alternations such as increased fasting glucose, triglyceride, total cholesterol and low-density lipoprotein (LDL) levels and decreased high-density lipoprotein (HDL) level [4–8].

In selected studies, breast cancer patients experience weight gain after initiation of adjuvant chemotherapy [4, 9–11]. Weight gain can, in turn, increase secretion of pro-inflammatory adipocytokines such as tumor necrosis factor α (TNF-α) and interleukin 6 (IL-6), causing local effects on white adipose tissue (e.g. lipid metabolism and insulin signaling) but also systemic effects on other organs [12]. Thus, treatment-induced weight gain results in increased inflammation, which ultimately increase the risk for type 2 diabetes and cardiovascular disease (CVD) [13–15]. Indeed, breast cancer survivors suffer from a significantly greater incidence of CVD such as heart failure and cardiac arrhythmias compared to the general female population [16, 17]. The most common cause of noncancer death for women with a history of breast cancer is CVD [18] and older women (> 66 years) diagnosed with EBC who survived five years or more after diagnosis are more likely to die from CVD than from the carcinoma itself [19].

Chemo- and radiotherapy have been designed to eliminate malignant cells, but they also significantly impact the immune system. Cytokines are crucial in controlling growth and activity of blood and other immune system cells. Plasma levels of cytokines have previously been measured in breast cancer patients to characterize breast cancer subtypes or to search for biomarkers of prognosis or treatment effect [20–22]. In addition, increased plasma levels of pro-inflammatory cytokines such as IL-6 and IL-8 has been shown during and after adjuvant radio- [23–26] and chemotherapy [24, 27, 28] in breast cancer patients. Studies with longer follow-ups are however sparse and results are conflicting [24, 29]. C-reactive protein (CRP), which is often used as a systemic marker of inflammation, is elevated in breast cancer patients treated with adjuvant chemotherapy in some [30] but not all [24, 31] studies. Knowledge about how chemo- and radiotherapy impact cytokine and CRP levels in the short and long term is important for understanding the development of inflammation-related comorbidities. In addition, it may have implications for immunotherapy and breast cancer recurrence rates. Indeed, higher IL-6 levels at diagnosis has been significantly associated with an increased risk of distant recurrence in patients with HER2-negative EBC treated with adjuvant chemotherapy [32].

We aimed to investigate changes of pro-inflammatory cytokine and CRP plasma levels before and shortly after adjuvant chemo- and radiotherapy treatment in 51 postmenopausal EBC patients. Furthermore, we aimed to examine if these changes are reversible during a 2-year follow-up. We also compared the 51 EBC patients with 41 age- and BMI-matched healthy controls to elucidate if breast cancer itself had an influence on pro-inflammatory mediators. Lastly, we related cytokine levels to metabolic parameters in the patients (e.g. plasma cholesterol, glucose, and insulin levels) as inflammation is closely related to metabolic diseases.

## Methods

### Study design, participants and sample collection

**Fig. 1:**
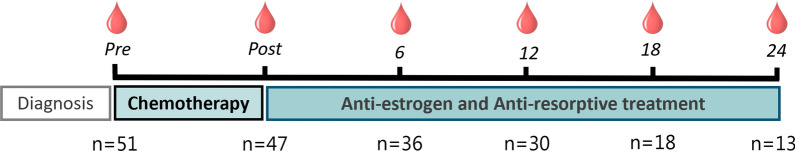
Study design. Early breast cancer patients were included in the clinical trial before initiation of chemotherapy treatment (Pre) and examined again shortly after (Post) and 6, 12, 18 and 24 months after completing chemotherapy. The number of patients included at each visit is indicated. 41 healthy controls matched for age and BMI were also included in the study, not visualized in this figure

In this study, we included 51 postmenopausal women aged 50–70 years with early (nonmetastatic, stage I-III) breast cancer eligible for chemotherapy, who had plasma samples available both before and after adjuvant chemotherapy by April 2022. The EBC patients are part of the project “Healthy living after breast cancer” at the Department of Oncology, Rigshospitalet [5]. Exclusion criteria included prior malignancy and preexisting endocrine disease. Recruitment and initial blood sampling occurred shortly before the initiation of adjuvant chemotherapy (Pre). Shortly after chemotherapy completion (median of 84 days) with (n = 41) or without (n = 10) radiotherapy, patients were examined at the Department of Endocrinology, Rigshospitalet (Post). After that, patients were examined at regular follow-up visits every six months up to two years after the Post visit (Fig. 1).

Information on tumor characteristics and oncological treatments of the 51 EBC patients are presented in Supplementary Table 1. The chemotherapy drugs paclitaxel, cyclophosphamide and epirubicin were given in combination to most of the patients (84%) for a median of 120 days. Out of the 51 patients, 26 had dose reductions or premature stops in their adjuvant chemotherapy regimens*.* All patients received higher-dose corticosteroids (methylprednisolone or prednisolone) during 2 or 3 days per chemotherapy cycle, to prevent side-effects from the chemotherapy treatment [33]. Twelve patients received neoadjuvant chemotherapy, and twenty patients received Trastuzumab treatment. Eighty percent (n = 41) of all patients received radiotherapy sequentially to chemotherapy. Forty-two patients had estrogen receptor-positive disease and 41 of them received anti-estrogen treatment (40 with aromatase inhibitors [Letrozole 2,5 mg/day] and one with Tamoxifen [20 mg/day]) after their chemotherapy treatment. Forty-five patients received zoledronic acid (4 mg) after completing chemotherapy (intravenous administration every six months; Supplementary Table 1). None of the included patients had triple-negative breast cancer and none received immunotherapy or poly (ADP-ribose) polymerase (PARP) inhibitors.

At each patient visit, K_2_EDTA plasma was collected and frozen at −80 °C. Key endocrine and metabolic parameters were analyzed in fasting plasma samples at the Department of Clinical Biochemistry, Rigshospitalet. In parallel, five healthy postmenopausal women were recruited to the study “Healthy living after breast cancer”. They had no endocrine or malignant diseases and did not use medication known to affect the metabolism. In addition, fasting plasma samples from 36 age- and BMI-matched postmenopausal healthy controls from a previous study (H-19034238) were included. These control patients were recruited to a different clinical study to investigate the effect of physical activity on various metabolic parameters in older untrained or trained, lean or obese, healthy women, where the clinical results previously have been published [34].

### MSD® V-plex pro-inflammatory cytokine assay preparation and procedure

The concentration of each cytokine in K_2_EDTA plasma was measured using the pre-coated, target-specific multiplex assay V-PLEX® Pro-inflammatory Panel 1 (human) kit (Meso Scale Discovery (MSD), Rockville, MD, USA). A seven-point standard curve with fourfold serial dilution steps was prepared, in double determinations for each plate, along with a zero-calibration blank.

K_2_EDTA plasma samples from patients and controls were thawed and pipetted into a 96-well plate. The plate was centrifuged at 2,000 × g for 3 min to remove particulates before dilution. The sample preparation and MSD analysis was done according to the manufacturer’s instruction, with the first plate analyzed in duplicates and the remaining three plates analyzed in single replicates. The sample concentrations were calculated using DISCOVERY WORKBENCH® 4.0 analysis software (MSD) and a 4-parameter logistic regression curve. Samples with undetectable cytokine levels were excluded. Samples with calculated cytokine concentrations below the lower limit of detection (LLOD) for each plate were imputed to ½ of the LLOD value for each plate.

### Evaluation of V-PLEX® Pro-inflammatory Panel 1 (human) kit data

The first 96-well plate was run in duplicates to evaluate the coefficient of variation (CV) in addition to the detection range. For IL-6, IL-8, IL-10, interferon γ (IFN-γ) and TNF-α, the CV was low (< 10%) for most samples, and more than 90% of all samples were in the detection range (Supplementary Table 2). Due to a high frequency of samples with high CV (above 20%) and non-detectable (too low) values for IL-1β, IL-2, IL-4, IL-12p70 and IL-13, these cytokines were evaluated to not give sufficiently reliable data in single runs. In fact, 23–78% of the samples had levels that were undetectable or below the LLOD for these cytokines, and 16–55% of the samples had a CV above 20% (Supplementary Table 2). This evaluation is in accordance with the manufacturer’s protocol, where the percentage of samples within the detection range for these cytokines in human EDTA plasma ranged from 4% (IL-13) to 37% (IL-1β) in their test run [35]. Based on this evaluation, it was decided to proceed with the analysis of IL-6, IL-8, IL-10, IFN-γ and TNF-α plasma levels only.

### hsCRP measurements

High-sensitivity CRP (hsCRP) values were measured in K_2_EDTA plasma on a Cobas® 6000 analyzer with the c501 module (Roche, Basel, Switzerland) using the kit Cardiac C-Reactive Protein (Latex) High-Sensitive (Roche). Before running the samples, the Cobas® 6000 analyzer was calibrated, and controls were tested [36]. Plasma samples from EBC patients and healthy controls were thawed, vortexed, centrifuged and pipetted into sample tubes, placed in sample racks and loaded onto the analyzer in single replicates. hsCRP values with concentrations below the LLOD were imputed to the LLOD value (0.15 mg/L).

### Statistical analyses

Statistical analysis and visualization were performed using GraphPad Prism statistical software v. 9.5.1 and R-4.2.2. The data distributions were analyzed using histograms, D’Agostino-Pearson and Kolmogrov-Smirnov tests for normality. If normally distributed, data are presented as mean and standard deviation (SD) whereas if not normally distributed, data are presented as median and interquartile range (IQR). The impact of chemotherapy on cytokine levels (Post vs. Pre) was assessed with Wilcoxon matched-pairs signed-rank tests. These tests were not corrected for multiple comparisons. Mann–Whitney tests were applied to assess differences between healthy controls and EBC patients. The percentage change with chemotherapy is given as the mean or median of difference (Pre to Post) relative to the Pre value. Since having missing values within the data (e.g., due to patients not attending some of the study appointments), the changes in cytokine levels during the two years after chemotherapy completion (e.g. 6-, 12-, 18- and 24-months visits vs Post) were analyzed with mixed models in R (lmer) with patient ID as random factor and study visit as fixed factor. Data was ln-transformed prior to the analysis. Proportions between groups were analyzed with two-proportions z-tests (prop.test in R).

For clinical data, Student’s unpaired (controls vs patients) and paired (patients at Pre vs Post visit) t-tests were used to test differences of parametric data, whereas Mann–Whitney and Wilcoxon tests were used for non-parametric data. The percentage differences between groups are given for differences between means (parametric data) and medians (non-parametric data), respectively. A mixed effects model with multiple comparisons was used to test changes between the Post visit and the 6-, 12-, 18-, and 24-month visits, respectively. Patients who were not in a fasted state (Pre: n = 3, Post: n = 3, 6 months: n = 5, 12 months: n = 1, 18 months: n = 4, 24 months: n = 2) were excluded from statistical analysis of glucose, insulin, HOMA-IR, C-peptide, LDL, and triglyceride levels. *P*-values of ≤ 0.05 were regarded as statistically significant.

## Results

### Worsened metabolic profile after adjuvant chemotherapy for EBC—with partial reversibility

Before initiation of chemotherapy, EBC patients had significantly higher fasting plasma glucose (5%, *p* = 0.047) and insulin (63%, *p* = 0.003) levels than age- and BMI-matched healthy controls. Notably, based on the HOMA-IR index, insulin resistance was 77% higher in EBC patients than in controls (*p* = 0.002). In addition, total plasma cholesterol (11%, p = 0.007) and LDL cholesterol (16%, *p* = 0.007) were significantly higher in EBC patients than in the controls (Table 1).

**Table 1:** Metabolic characteristics of EBC patients and healthy controls. Metabolic characteristics of EBC patients before (Pre), shortly after (Post), and 6, 12, 18 and 24 months after completed adjuvant chemotherapy as well as of 41 age- and BMI-matched healthy controls

	Controls n = 41	EBC Pre n = 51	EBC Post n = 47	EBC 6 months n = 36	EBC 12 months n = 30	EBC 18 months n = 18	EBC 24 months n = 18
Age (years)	58 ± 4	59 ± 5	N/A	N/A	N/A	N/A	N/A
Weight (kg)	75.8 ± 19.2	76.6 ± 13.2	77.6 ± 14.6	N/A	76.7 ± 12.7	N/A	77.1 ± 13.6
BMI (kg/m^2^)	27.4 ± 6.3	27.8 ± 5.2	27.9 ± 5.4	N/A	27.6 ± 5.6	N/A	28.1 ± 6.2
Glucose (mmol/L)	5.4 ± 0.59	5.6 ± 0.7¤	5.7 ± 1.1	5.5 ± 1.0	5.3 ± 0.68#	5.4 ± 0.45	5.5 ± 0.46
Insulin (pmol/L)	39 (30, 60)	64 (40, 101)¤¤	81 (63, 114)*	84 (51, 100)	76 (58, 95)	54 (46, 92)#	57 (41, 102)
HOMA-IR	1.5 (1.2, 2.5)	2.7 (1.6, 4.2)¤¤	3.2 (2.4, 5.3)	3.0 (1.9, 4.2)	2.9 (2.2, 3.5)	2.2 (1.8, 3.5)#	2.2 (1.6, 4.1)
C-peptide (pmol/L)	N/A	782 (638, 1098)	875 (722, 1175)	824 (702, 927)#	799 (669, 949)	752 (651, 851)	707 (594, 859)
HbA1c (mmol/mol)	37 (33, 39)	35 (34, 38)	36 (34, 38)	36 (35, 38)	38 (36, 39)	37 (35, 39)	36 (35, 39)
Total cholesterol (mmol/L)	5.3 ± 0.66	5.8 ± 1.1¤	5.9 ± 1.0	6.0 ± 1.1	5.8 ± 0.97	6.1 ± 1.3	5.8 ± 1.5
HDL (mmol/L)	1.9 ± 0.48	1.8 ± 0.46	1.6 ± 0.38**	1.8 ± 0.37#	1.8 ± 0.42#	1.8 ± 0.31#	1.8 ± 0.38
LDL (mmol/L)	3.2 ± 0.63	3.7 ± 1.0¤	3.8 ± 0.94	3.9 ± 0.86	3.7 ± 0.93	3.8 ± 1.2	3.6 ± 1.4
Triglycerides (mmol/L)	1.1 ± 0.62	1.2 ± 0.56	1.5 ± 0.73*	1.3 ± 0.48	1.2 ± 0.45	1.2 ± 0.36	1.3 ± 0.44
Hemoglobin (mmol/L)	N/A	8.4 ± 0.65	8.0 ± 0.50**	8.1 ± 0.58	8.2 ± 0.46#	8.3 ± 0.40#	8.4 ± 0.45#
Leucocytes (10^9^/L)	N/A	5.8 ± 1.4	4.7 ± 1.3**	4.6 ± 1.1	4.9 ± 1.4	4.9 ± 0.80	5.4 ± 1.6

Data are presented as mean ± SD for parametric variables and median (25%, 75% interquartile range) for non-parametric variables. Patients who were not in a fasted state (Pre: n = 3, Post: n = 3, 6 months: n = 5, 12 months: n = 1, 18 months: n = 4, 24 months: n = 2) were excluded from analysis of glucose, insulin, HOMA-IR, C-peptide, LDL, and triglycerides. Significantly different from controls: ¤*p* < 0.05; ¤¤*p* < 0.005. Significantly different from Pre visit: *p < 0.05; **p < 0.005. Significantly different from Post visit: #*p* < 0.05. HOMA-IR: Homeostatic model assessment for insulin resistance. HbA1c: Hemoglobin A1c. HDL: High-density lipoprotein. LDL: Low-density lipoprotein. hsCRP: high-sensitivity C-reactive protein. N/A: Not applicable. Pre, before chemotherapy. Post, after chemotherapy

In the EBC patients, weight and BMI were not significantly different after vs before chemotherapy treatment (Table 1). However, insulin levels increased by 27% (*p* = 0.017) and the HOMA-IR index increased by 21% (*p* = 0.0504) after vs before chemotherapy in the patients. Triglyceride levels increased significantly (19%, *p* = 0.008) whereas HDL cholesterol decreased (21%, *p* < 0.0001), being in line with our previous publication (5). Lastly, hemoglobin and leukocyte levels decreased by 5% (*p* = 0.020) and 20% (*p* < 0.0001), respectively (Table 1).

During the first year after chemotherapy, plasma insulin levels and insulin resistance remained high in the EBC patients. Eighteen months after the completion of chemotherapy, insulin and HOMA-IR were significantly decreased (both *p* < 0.05) compared to shortly after chemotherapy (Post). Plasma glucose levels were significantly reduced 12 months after ended chemotherapy compared to at the Post visit (5.3 mmol/L vs 5.7 mmol/L, *p* = 0.007) and HDL cholesterol was significantly increased both 6, 12 and 18 months after the Post visit (all 1.8 mmol/L vs. 1.6 mmol/L at Post visit; Table 1). Hemoglobin increased to pre-adjuvant therapy levels 12 months after completing chemotherapy and remained stable (*p* < 0.05 at 12-, 18- and 24-month visits vs the Post visit, Table 1).

### Transient increase of pro-inflammatory cytokines after adjuvant chemotherapy treatment

Ten pro-inflammatory cytokines were measured in all available plasma samples from the 51 EBC patients and the 41 healthy controls using V-PLEX Pro-inflammatory Panel 1 (MSD). Because of low, undetectable levels and high CV for five of the cytokines (IL-1β, IL-2, IL-4, IL-12p70 and IL-13; see methods), only data on IL-6, IL-8, IL-10, IFN-γ and TNF-α are presented here.

**Fig. 2:**
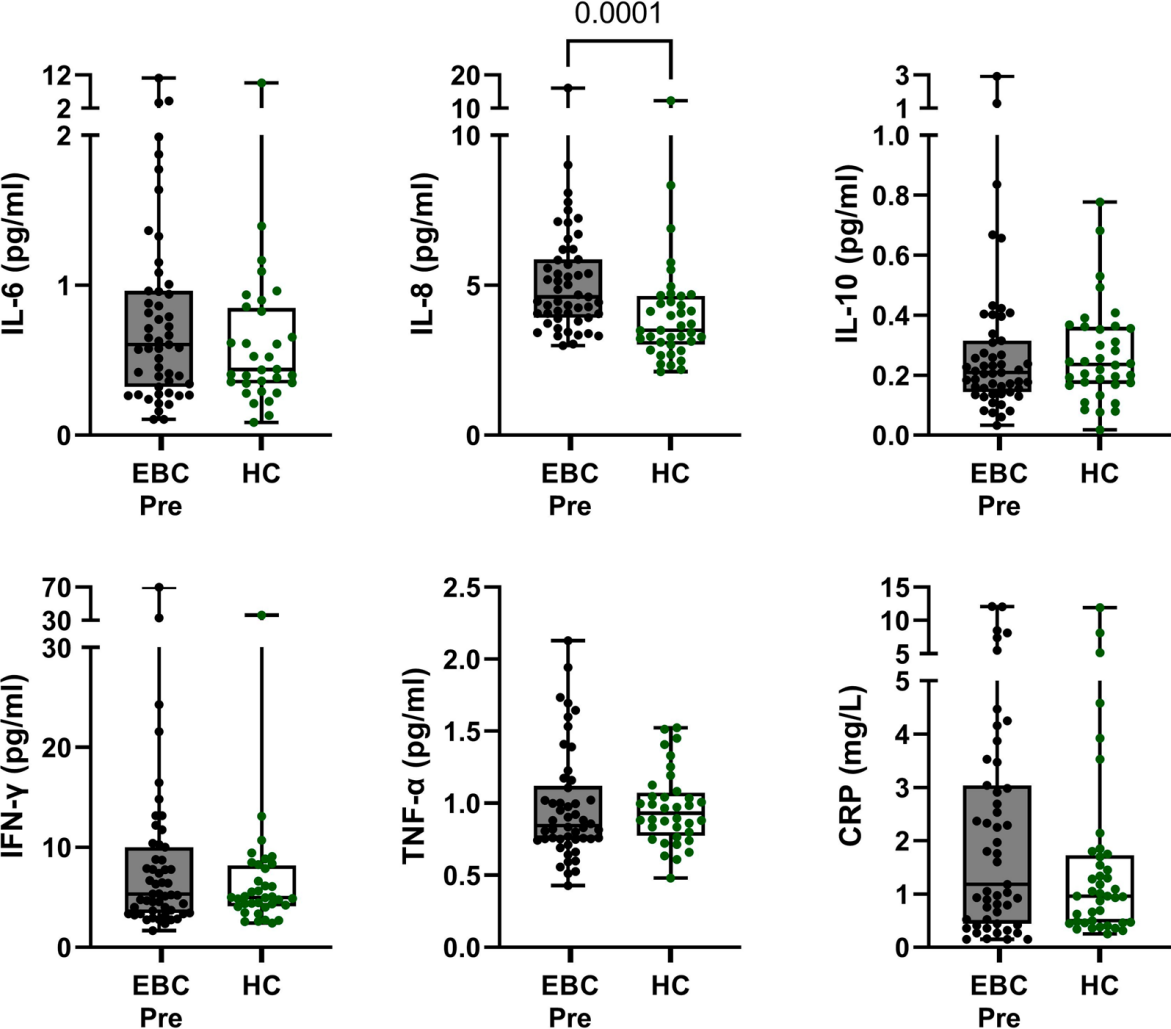
Cytokine and hsCRP levels in 51 EBC patients before chemotherapy vs 41 healthy controls. Data are presented by boxplots indicating medians, interquartile range (25th to 75th percentile) and minimum to maximum (whiskers). Differences between groups were analyzed with Mann–Whitney tests. EBC, early breast cancer. hsCRP, high-sensitivity C-reactive protein. HC, healthy controls. Pre, before chemotherapy

First, differences in cytokine levels between EBC patients and healthy controls were studied. No significant differences existed in plasma cytokine concentration of IL-6, IL-10, IFN-γ or TNF-α between EBC patients before chemotherapy treatment and healthy controls. However, plasma IL-8 levels were significantly higher in EBC patients compared to the controls (31%, *p* = 0.0001; Fig. 2). IL-8 levels were not different in patients who had (n = 39) or had not (n = 12) undergone a tumor resection at the time of blood sampling (*p* = 0.24), and both groups had significantly higher IL-8 levels compared to the controls (Supplementary Fig. 1).

**Fig. 3:**
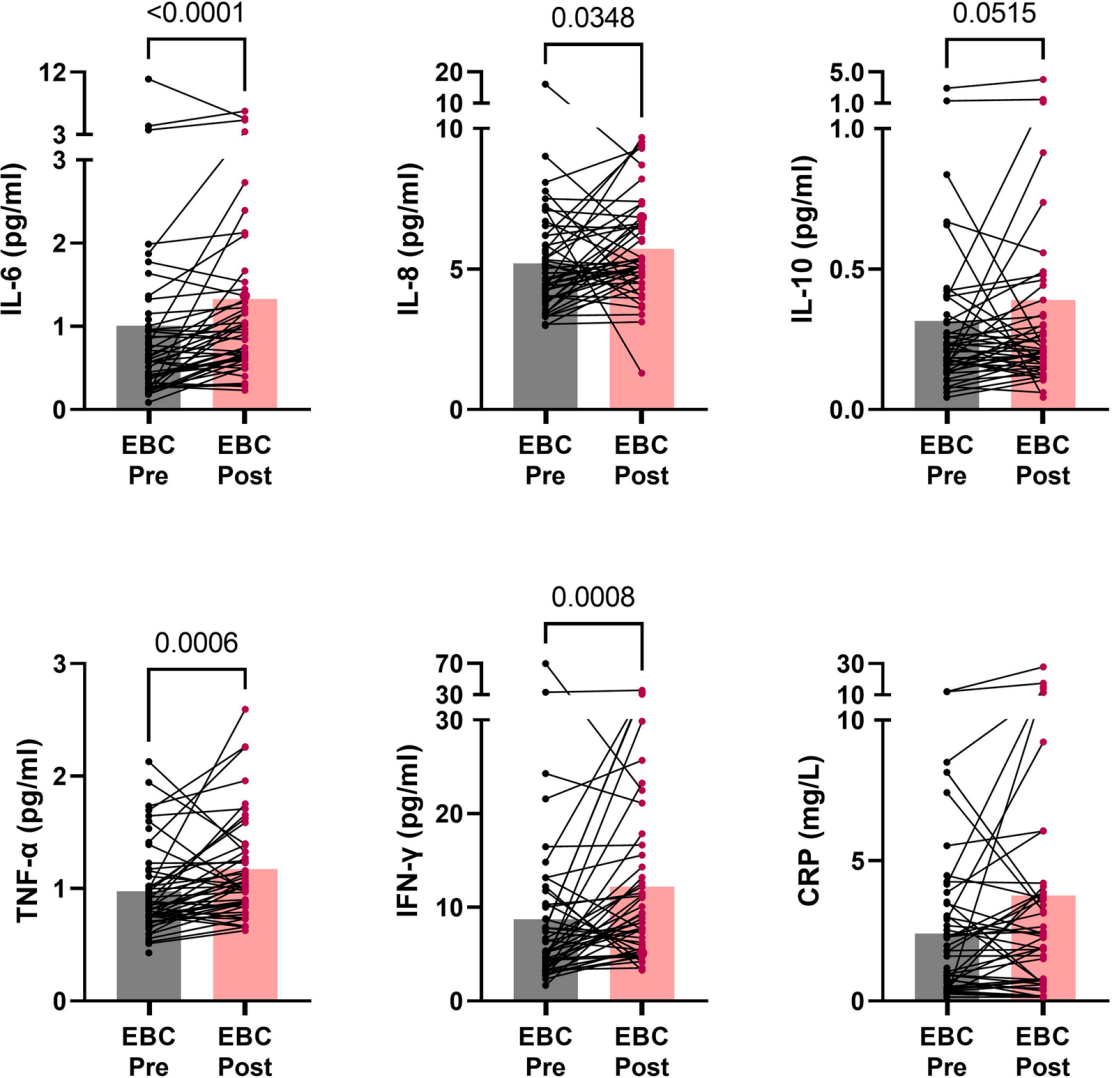
Cytokine and hsCRP levels in EBC patients before versus after chemotherapy. 51 EBC patients were investigated before (Pre, grey bars) and 47 of them after (Post, pink bars) chemotherapy. Differences between groups were examined by Wilcoxon tests. EBC, early breast cancer. hsCRP, high-sensitivity C-reactive protein. Pre, before chemotherapy. Post, after chemotherapy

In the EBC patients, significantly increased levels of IL-6 (39%, *p* < 0.0001), IL-8 (15%, *p* < 0.0001), IL-10 (20%, *p* = 0.0515), IFN-γ (48%, *p* = 0.0008) and TNF-α (16%, *p* = 0.0006) were detected shortly after vs before chemotherapy (Fig. 3). Cytokine levels were not different in patients who had received neoadjuvant compared to adjuvant chemotherapy and both groups had significantly or borderline significantly higher levels of IL-6, IL-8 and IFN-γ shortly after chemotherapy compared to the healthy controls (Supplementary Fig. 2).

**Fig. 4:**
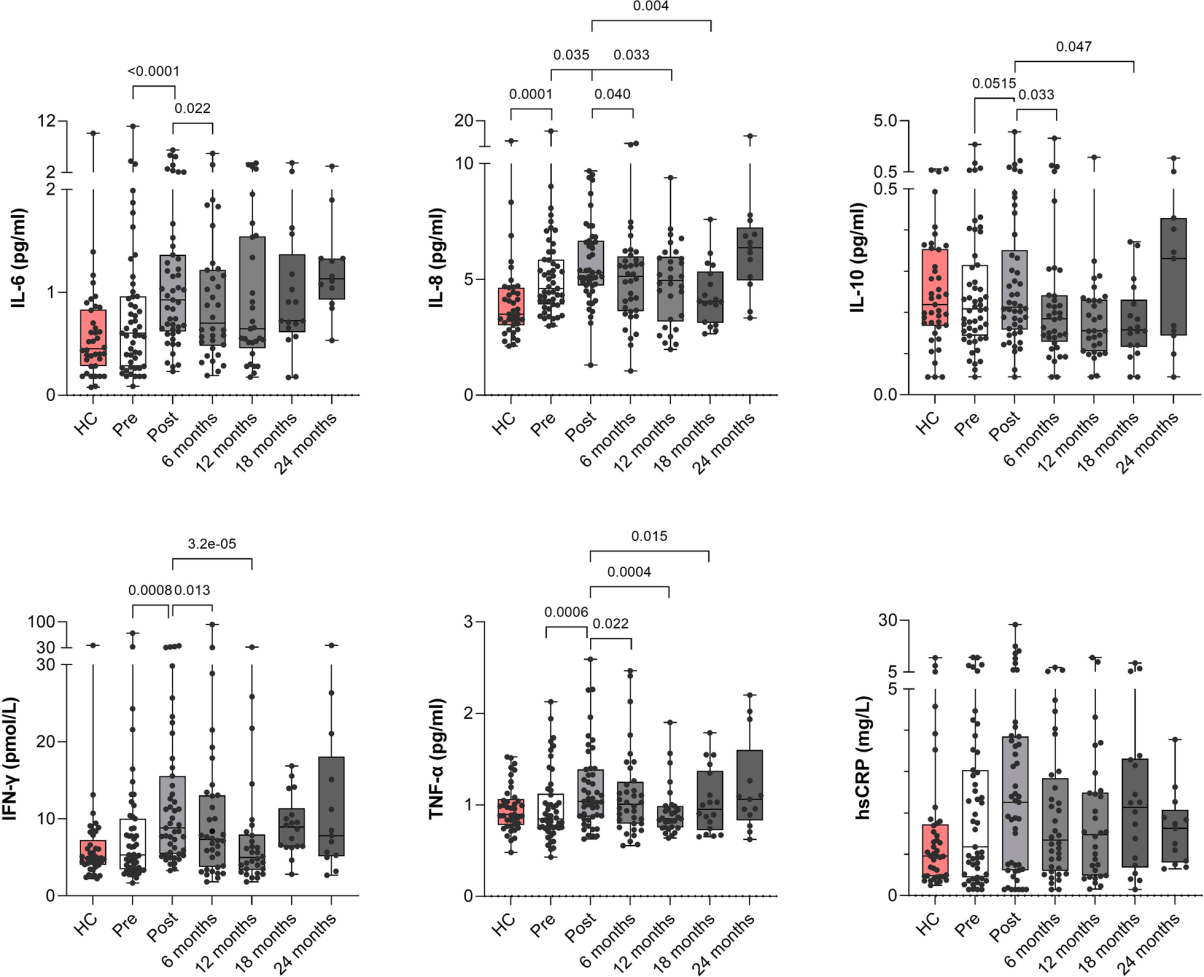
Cytokine and hsCRP fluctuations before and during two years after adjuvant chemotherapy for EBC. Cytokines levels were measured for all plasma samples that were available from EBC patients in the clinical trial by April 2022 (Pre: n = 51, Post: n = 47; 6 months: n = 36, 12 months: n = 30, 18 months: n = 18 and 24 months: n = 13) and from 41 healthy controls. Boxplots display data indicating median values, interquartile range (25th to 75th percentile) and minimum to maximum (whiskers). *P*-values are based on Wilcoxon tests (Pre vs Post) and linear mixed models (Post vs. 6, 12, 18 and 24 months) of ln-transformed data. Of note, all available data are presented in the figures, whereas statistical tests are based on paired data only. EBC, early breast cancer. HC, healthy controls. hsCRP, high-sensitivity C-reactive protein. Pre, before chemotherapy. Post, after chemotherapy

The effect of time (6, 12, 18 and 24 months) on cytokine levels after ended chemo- and radiotherapy treatment was then analyzed. Most patients (76%) received anti-estrogen and anti-resorptive treatment during this time, which had been initiated at the Post visit. Interestingly, all cytokine levels significantly decreased six months after chemotherapy treatment compared to shortly after chemotherapy (Post; all *p* < 0.05; Fig. 4). In line with this, all cytokines had significantly (IL-8, IFN-γ, TNF-α) or borderline significantly (IL-6, *p* = 0.096; IL-10, *p* = 0.069) decreased levels 12 months after ended chemotherapy compared to the Post visit (Fig. 4). Cytokine levels 6 or 12 months after chemotherapy completion were similar to pre-chemotherapy levels. Thus, the observed increase in pro-inflammatory cytokines was reversed during the first year after chemotherapy treatment in EBC patients. For patients who were examined 18 months after chemotherapy ended, IL-8, IL-10 and TNF-α were significantly lower than at the Post visit (Fig. 4).

Thirteen of the EBC patients had been examined 24 months after ending chemotherapy. Individual changes in cytokine levels before and during 24 months after chemotherapy are illustrated in Supplementary Fig. 3. Although the variations in cytokine levels are large for some patients, most patients have a similar cytokine level 24 months after compared to before chemotherapy.

In our study population, all patients received chemotherapy and 41 of the 51 EBC patients received radiotherapy treatment. Four patients did neither receive radio- nor anti-estrogen therapy. Another six patients received radiotherapy but no anti-estrogens. In Supplementary Fig. 4, we illustrate how cytokine levels change with adjuvant therapies in (1) 35 patients who received chemo-, radio and anti-estrogen therapy, (2) 6 patients who received chemo- and radiotherapy, (3) 6 patients who received chemo- and anti-estrogen therapy, and (4) 4 patients who received chemotherapy only. Due to the small number of patients in each group, the impact of radio- and/or anti-estrogen therapy in addition to chemotherapy on plasma cytokines levels in EBC patients is difficult to interpret.

### A large proportion of patients have high hsCRP levels

There was no significant difference in plasma hsCRP levels between EBC patients before chemotherapy and controls (Fig. 2). However, a larger proportion of the patients had a hsCRP level above 2 mg/L (25 of 51 patients (41%), 7 of 41 controls (17%); Χ^2^ = 5.15, *p* = 0.023). The plasma hsCRP level of EBC patients increased from a median level of 1.8 mg/L before to a median level of 2.3 mg/L after chemotherapy. After chemotherapy, 51% of the patients had a hsCRP level above 2 mg/L and 38% above 3 mg/L (Fig. 3). Plasma hsCRP levels showed a significant correlation with IL-6 levels (Supplementary Table 3) and borderline significant associations with IFN-γ (r = 0,26, *p* = 0.065) and TNF-α (r = 0,26, *p* = 0.070) levels in the EBC patients before chemotherapy.

### Altered lipid profile after chemotherapy may contribute to the rise in plasma IL-6, IFN-γ, TNF-α and hsCRP levels

Because inflammatory cytokines can influence the metabolism and blood lipids can contribute to inflammatory processes, the EBC patients’ cytokine and hsCRP levels were associated with clinical characteristics that significantly changed with chemotherapy in the EBC patients (Table 1 and Supplementary Table 4). Notably, IL-6 correlated significantly with all the clinical parameters that significantly changed with chemotherapy in the patients (fasting insulin, HDL cholesterol, triglycerides, hemoglobin and leukocytes; Supplementary Table 4). Also hsCRP, which intercorrelated with IL-6 levels (Supplementary Table 3), correlated significantly with all parameters except HDL cholesterol and hemoglobin. Plasma IL-10 showed no significant associations, whereas IL-8 correlated positively with hemoglobin levels, IFN-γ with triglyceride levels and TNF-α correlated negatively with HDL cholesterol (Supplementary Table 4). Thus, the increase in triglyceride levels and the decrease in HDL levels observed after chemotherapy may partly contribute to the increase in IL-6, IFN-γ, TNF-α and hsCRP levels after chemotherapy.

## Discussion

In this study, we aimed to investigate if adjuvant chemotherapy with or without radiotherapy alters the plasma level of five inflammatory cytokines and hsCRP in postmenopausal EBC patients, and whether those changes persisted or were reversed to pre-adjuvant treatment levels up to two years after ended chemotherapy treatment. Our study shows significant increases in plasma concentrations of the pro-inflammatory cytokines IL-6, IL-8, IFN-γ and TNF-α and the anti-inflammatory cytokine IL-10, but not hsCRP, after adjuvant chemotherapy. Already six months after chemotherapy completion, all plasma cytokine levels were significantly reduced. In fact, the cytokine levels at the 6- or 12-month visit after chemotherapy are comparable to the pre-chemotherapy level. Thus, our study indicates that plasma levels of pro-inflammatory cytokines return to pre-treatment levels within the first year after undergoing chemo- and radiotherapy in postmenopausal EBC patients.

In line with our results, other longitudinal studies have shown increased levels of IL-6 [24, 27–29], IL-8 [24, 27], IFN-γ [24] and TNF-α [24], with no significant change in hsCRP [24, 31], in EBC patients receiving chemo- and/or radiotherapy. Few studies have followed breast cancer patients for more than 1–2 years to study the long-term effects of chemotherapy on inflammatory markers. In the most thorough study so far, Bower et al. followed pre- and postmenopausal breast cancer patients up to 18 months after treatment with either chemotherapy, radiotherapy, both or none [24]. Authors observed increased levels of IL-6, IL-8, IFN-γ and TNF-α in patients receiving chemotherapy with or without radiotherapy, although the rise in IL-8 was first observed six months after chemotherapy completion in patients who only received chemotherapy [24]. In contrast to our study, plasma levels of IL-6, IL-8, IFN-γ and TNF-α were increased 6, 12 and 18 months after chemo- and radiotherapy completion compared to before initiating these adjuvant therapies [24]. In a study by Lyon et al., chemotherapy-induced levels of IL-6 (but not IL-8, IL-10 and TNF- α) dropped to below pre-treatment levels over a 2-year follow-up in pre- and postmenopausal EBC patients [29]. In this and our study, a larger proportion of patients received anthracyclines (86% receive epirubicin in our study, 55% receive doxorubicin in the Lyon et al. study) compared to the Bower et al. study where only 9% of patients received doxorubicin. It is not clear whether the different chemotherapy regimens may impact the inflammatory markers differently over the 2-year follow-up. Finally, all EBC patients were administered corticosteroids as part of their chemotherapy regimens. Because corticosteroids are known to reduce the systemic inflammatory response [37], they most likely did not cause the chemotherapy-induced increase in cytokine levels.

Inflammation is closely related to insulin resistance, dyslipidemia, and obesity. In our patient cohort and in other studies, insulin resistance, hyperinsulinemia, and dyslipidemia (low HDL cholesterol, high LDL cholesterol and triglycerides) were noticeable in patients vs controls already before initiation of adjuvant therapies and these became even more pronounced after the chemotherapy completion. Because IL-6, IFN-γ, TNF-α and hsCRP levels correlate significantly with triglycerides, HDL or both in the patients, we speculate that therapy-induced alterations to the lipid profile may partly contribute to the changes in hsCRP and plasma cytokine levels observed after chemotherapy. On the other hand, pro-inflammatory cytokines could influence lipid metabolism by affecting adipose tissue, liver, and other organs involved in lipid regulation, and thereby increase the risk of cardiometabolic disease. It would be interesting to investigate whether treatment with statins could decrease the degree of inflammation seen post chemotherapy.

C-reactive protein is an acute phase protein produced in the liver in response to IL-6. Even though its response to adjuvant therapy was not significant in our study or other similar studies [24, 31], a larger proportion of patients (41% before and 51% after chemotherapy) than the controls (7%) had hsCRP levels above 2 mg/L. Indeed, hsCRP levels above 2 mg/L are considered metabolic inflammation, and high levels of hsCRP are associated with increased risks of type 2 diabetes [38] and CVD [39, 40], especially if combined with elevated levels of total and LDL cholesterol, triglycerides and glucose. Raised hsCRP levels in the patients might partly be due to the inflammatory response caused by tumor resection in the patients. Six and 12 months after ending chemotherapy, hsCRP levels of most EBC patients are comparable to the levels of the age- and BMI-matched healthy controls.

Before initiation of chemotherapy, the EBC patients exhibit higher concentrations of plasma IL-8 compared to the healthy controls, which has been shown in previous studies [41–43]. IL-8 is a chemoattractant with a key role in attracting and activating neutrophils at sites of inflammation [44] and tumor lesions [45]. It has been shown that IL-8 expression is higher in breast cancer cells from high-stage vs low-stage patients and that adipose-derived stem cells isolated from breast tissue in patients express two-fold more IL-8 compared to controls [46]. We did not observe a significant difference in IL-8 plasma levels in patients with or without a tumor present at the pre-chemotherapy blood sampling. This might indicate that adipose-derived cells in the breast are responsible for the increased IL-8 plasma levels in postmenopausal EBC patients.

Compelling evidence indicates that both chemo- and radiotherapy activate inflammatory pathways at a systemic level and within the tumor microenvironment [47, 48]. We reinforce the importance of immune cell activation in response to chemo- and radiotherapy by showing increased mitochondrial respiratory capacity together with an increased mitochondrial DNA content in mononuclear blood cells from EBC patients [49]. Moreover, even if the leukocyte numbers significantly decrease in the current study, the patients’ immune cells are more active and release increased levels of cytokines during the first weeks after chemotherapy completion. The release of e.g. IL-6 from subcutaneous adipose tissue may also contribute to the chemotherapy-related increase [50]. As seen here, the inflammatory response is temporary and reversed to pre-treatment levels during the following 6–12 months, which may signify elimination of senescent cells and bone marrow microenvironment restoration during the first few months after radio- and chemotherapy treatment.

## Conclusions

The results of this study indicate that adjuvant chemo- and radiotherapy cause increased concentrations of plasma pro-inflammatory cytokines together with dyslipidemia and insulin resistance in postmenopausal women with EBC. The rise in cytokine levels following anthracycline and taxane based adjuvant chemotherapy is temporary, with levels significantly returning to pre-treatment values for most patients six months after completing chemotherapy. Even so, the therapy-induced rise in pro-inflammatory cytokine levels together with the insulin resistance and worsened lipid profile seen in the patients may promote inflammation in metabolically important tissues as well as the cardiovascular system, thus contributing to the development of metabolic syndrome, type 2 diabetes and CVD in postmenopausal EBC survivors. These potential long-term consequences from the upregulation of inflammatory reactions after chemotherapy should be further studied to understand its role in cardiometabolic disease development in breast cancer survivors.

## Supplementary Information

Additional file 1.

## Data Availability

The datasets used and analyzed during the current study are available from the corresponding author on reasonable request.

## Abbreviations

BMI: Body mass index
CVD: Cardiovascular disease
EBC: Early breast cancer
hsCRP: High-sensitivity C-reactive protein
HDL: High-density lipoprotein
HbA1c: Hemoglobin A1c
HOMA-IR: Homeostatic model assessment for insulin resistance
IL: Interleukin
LDL: Low-density lipoprotein
Pre: Before chemotherapy
Post: Shortly after chemotherapy
TNF-α: Tumor necrosis factor α

## References

[1] Sung H, Ferlay J, Siegel RL, Laversanne M, Soerjomataram I, Jemal A, Bray F. Global cancer statistics 2020: GLOBOCAN estimates of incidence and mortality worldwide for 36 cancers in 185 countries. CA Cancer J Clin. 2021;71:209–49. 33538338 10.3322/caac.21660

[2] Miller KD, Nogueira L, Mariotto AB, Rowland JH, Yabroff KR, Alfano CM, Jemal A, Kramer JL, Siegel RL. Cancer treatment and survivorship statistics, 2019. CA Cancer J Clin. 2019;69:363–85. 31184787 10.3322/caac.21565

[3] Altun İ, Sonkaya A. The most common side effects experienced by patients were receiving first cycle of chemotherapy. Iran J Public Health. 2018;47:1218–9. 30186799 PMC6123577

[4] Trédan O, Bajard A, Meunier A, et al. Body weight change in women receiving adjuvant chemotherapy for breast cancer: a French prospective study. Clin Nutr. 2010;29:187–91. 19713014 10.1016/j.clnu.2009.08.003

[5] Buch-Larsen K, Lund-Jacobsen T, Andersson M, Schwarz P. Weight change in post-menopausal women with breast cancer during chemotherapy-perspectives on nutrition, activity and bone metabolism: an interim analysis of a 5-year prospective cohort. Nutrients. 2021;13(8):2902. 10.3390/nu13082902. 34445061 10.3390/nu13082902 PMC8398627

[6] Servaes P, Verhagen C, Bleijenberg G. Fatigue in cancer patients during and after treatment: prevalence, correlates and interventions. Eur J Cancer. 2002;38:27–43. 11750837 10.1016/s0959-8049(01)00332-x

[7] Ryu HH, Ahn SH, Kim SO, et al. Comparison of metabolic changes after neoadjuvant endocrine and chemotherapy in ER-positive, HER2-negative breast cancer. Sci Rep. 2021;11:10510. 34006898 10.1038/s41598-021-89651-0 PMC8131718

[8] Kwan ML, Cheng RK, Iribarren C, et al. Risk of cardiometabolic risk factors in women with and without a history of breast cancer: the pathways heart study. J Clin Oncol. 2022;40:1635–46. 35025627 10.1200/JCO.21.01738 PMC9113213

[9] Dixon JK, Moritz DA, Baker FL. Breast cancer and weight gain: an unexpected finding. Oncol Nurs Forum. 1978;5:5–7. 248815

[10] Demark-Wahnefried W, Winer EP, Rimer BK. Why women gain weight with adjuvant chemotherapy for breast cancer. J Clin Oncol. 1993;11:1418–29. 8315439 10.1200/JCO.1993.11.7.1418

[11] Shapiro CL, Recht A. Side effects of adjuvant treatment of breast cancer. N Engl J Med. 2001;344:1997–2008. 11430330 10.1056/NEJM200106283442607

[12] Trayhurn P, Wood IS. Adipokines: inflammation and the pleiotropic role of white adipose tissue. Br J Nutr. 2004;92:347–55. 15469638 10.1079/bjn20041213

[13] Powell-Wiley TM, Poirier P, Burke LE, et al. Obesity and cardiovascular disease: a scientific statement from the american heart association. Circulation. 2021;143:e984–1010. 33882682 10.1161/CIR.0000000000000973 PMC8493650

[14] Ruparelia N, Chai JT, Fisher EA, Choudhury RP. Inflammatory processes in cardiovascular disease: a route to targeted therapies. Nat Rev Cardiol. 2017;14:133–44. 27905474 10.1038/nrcardio.2016.185 PMC5525550

[15] Donath MY, Shoelson SE. Type 2 diabetes as an inflammatory disease. Nat Rev Immunol. 2011;11:98–107. 21233852 10.1038/nri2925

[16] Mehta LS, Watson KE, Barac A, et al. Cardiovascular disease and breast cancer: where these entities intersect: a scientific statement from the american heart association. Circulation. 2018;137:e30–66. 29437116 10.1161/CIR.0000000000000556 PMC6722327

[17] Jakobsen M, Kolodziejczyk C, Jensen MS, Poulsen PB, Khan H, Kümler T, Andersson M. Cardiovascular disease in women with breast cancer - a nationwide cohort study. BMC Cancer. 2021;21:1040. 34537007 10.1186/s12885-021-08716-5 PMC8449438

[18] Afifi AM, Saad AM, Al-Husseini MJ, Elmehrath AO, Northfelt DW, Sonbol MB. Causes of death after breast cancer diagnosis: a US population-based analysis. Cancer. 2020;126:1559–67. 31840240 10.1002/cncr.32648

[19] Abdel-Qadir H, Austin PC, Lee DS, Amir E, Tu JV, Thavendiranathan P, Fung K, Anderson GM. A population-based study of cardiovascular mortality following early-stage breast cancer. JAMA Cardiol. 2017;2:88–93. 27732702 10.1001/jamacardio.2016.3841

[20] Jabeen S, Espinoza JA, Torland LA, et al. Noninvasive profiling of serum cytokines in breast cancer patients and clinicopathological characteristics. Oncoimmunology. 2019;8: e1537691. 30713794 10.1080/2162402X.2018.1537691 PMC6343793

[21] Kawaguchi K, Sakurai M, Yamamoto Y, et al. Alteration of specific cytokine expression patterns in patients with breast cancer. Sci Rep. 2019;9:2924. 30814616 10.1038/s41598-019-39476-9 PMC6393524

[22] Jabeen S, Zucknick M, Nome M, et al. Serum cytokine levels in breast cancer patients during neoadjuvant treatment with bevacizumab. Oncoimmunology. 2018;7:e1457598. 30377556 10.1080/2162402X.2018.1457598 PMC6205029

[23] Marconi R, Serafini A, Giovanetti A, Bartoleschi C, Pardini MC, Bossi G, Strigari L. Cytokine modulation in breast cancer patients undergoing radiotherapy: a revision of the most recent studies. Int J Mol Sci. 2019. 10.3390/ijms20020382. 10.3390/ijms20020382 PMC6359111 30658426

[24] Bower JE, Ganz PA, Irwin MR, et al. Acute and chronic effects of adjuvant therapy on inflammatory markers in breast cancer patients. JNCI Cancer Spectr. 2022. 10.1093/jncics/pkac052. 10.1093/jncics/pkac052 PMC9420043 35900175

[25] Aula H, Skyttä T, Tuohinen S, Luukkaala T, Hämäläinen M, Virtanen V, Raatikainen P, Moilanen E, Kellokumpu-Lehtinen P-L. Decreases in TGF-β1 and PDGF levels are associated with echocardiographic changes during adjuvant radiotherapy for breast cancer. Radiat Oncol. 2018;13:201. 30340644 10.1186/s13014-018-1150-7 PMC6194684

[26] Schmidt ME, Meynköhn A, Habermann N, et al. Resistance exercise and inflammation in breast cancer patients undergoing adjuvant radiation therapy: mediation analysis from a randomized, controlled intervention trial. Int J Radiat Oncol Biol Phys. 2016;94:329–37. 26853341 10.1016/j.ijrobp.2015.10.058

[27] Zhang Z, Ormiston K, Schnell P, Kopec R, Lustberg M, Orchard T. Changes in serum fatty acids and inflammatory markers in postmenopausal women with breast cancer following chemotherapy treatment. Curr Dev Nutr. 2021;5:290.

[28] Cheung YT, Ng T, Shwe M, et al. Association of proinflammatory cytokines and chemotherapy-associated cognitive impairment in breast cancer patients: a multi-centered, prospective, cohort study. Ann Oncol Off J Eur Soc Med Oncol. 2015;26:1446–51. 10.1093/annonc/mdv206 PMC4478978 25922060

[29] Lyon DE, Cohen R, Chen H, Kelly DL, McCain NL, Starkweather A, Ahn H, Sturgill J, Jackson-Cook CK. Relationship of systemic cytokine concentrations to cognitive function over two years in women with early stage breast cancer. J Neuroimmunol. 2016;301:74–82. 27890459 10.1016/j.jneuroim.2016.11.002 PMC5181109

[30] Dieli-Conwright CM, Wong L, Waliany S, Bernstein L, Salehian B, Mortimer JE. An observational study to examine changes in metabolic syndrome components in patients with breast cancer receiving neoadjuvant or adjuvant chemotherapy. Cancer. 2016;122:2646–53. 27219902 10.1002/cncr.30104 PMC4992442

[31] Hu JJ, Urbanic JJ, Case LD, et al. Association between inflammatory biomarker C-reactive protein and radiotherapy-induced early adverse skin reactions in a multiracial/ethnic breast cancer population. J Clin Oncol. 2018;36:2473–82. 29989859 10.1200/JCO.2017.77.1790 PMC6097833

[32] Sparano JA, O’Neill A, Graham N, Northfelt DW, Dang CT, Wolff AC, Sledge GW, Miller KD. Inflammatory cytokines and distant recurrence in HER2-negative early breast cancer. NPJ breast cancer. 2022;8:16. 35136076 10.1038/s41523-021-00376-9 PMC8825796

[33] Marina D, Buch-Larsen K, Gillberg L, Andersen MA, Andersson M, Rasmussen ÅK, Schwarz P. Chemotherapy for post-menopausal women with early breast cancer seems not to result in clinically significant changes in thyroid function. Cancer Med. 2024;13: e70015. 39108148 10.1002/cam4.70015 PMC11303825

[34] Frandsen J, Hansen IMD, Wismann JF, et al. Maximal fat oxidation rate is higher in fit women and unfit women with obesity, compared to normal-weight unfit women. J Clin Endocrinol Metab. 2021;106:e4389–99. 34185854 10.1210/clinem/dgab473

[35] Meso Scale Discovery. Proinflammatory panel 1 (human) Kits. V-PLEX. 2014;1:1–35.

[36] Cobas CRPHS. Roche 5

[37] Roxburgh CSD, McMillan DC. Cancer and systemic inflammation: treat the tumour and treat the host. Br J Cancer. 2014;110:1409–12. 24548867 10.1038/bjc.2014.90 PMC3960633

[38] Wang X, Bao W, Liu J, et al. Inflammatory markers and risk of type 2 diabetes. Diabetes Care. 2013;36:166–75. 23264288 10.2337/dc12-0702 PMC3526249

[39] Pearson TA, Mensah GA, Alexander RW, et al. Markers of inflammation and cardiovascular disease: application to clinical and public health practice: a statement for healthcare professionals from the centers for disease control and prevention and the american heart association. Circulation. 2003;107:499–511. 12551878 10.1161/01.cir.0000052939.59093.45

[40] Ridker PM, Hennekens CH, Buring JE, Rifai N. C-reactive protein and other markers of inflammation in the prediction of cardiovascular disease in women. N Engl J Med. 2000;342:836–43. 10733371 10.1056/NEJM200003233421202

[41] Zakrzewska I, Omyła J. The value of plasma interleukin-6 and interleukin-8 in monitoring of patients with breast cancer. Pol Merkur Lekarski. 2005;18:424–6. 16161926

[42] Mendonça MAO, Souto FO, Micheli DC, Alves-Filho JC, Cunha FQ, Murta EFC, Tavares-Murta BM. Mechanisms affecting neutrophil migration capacity in breast cancer patients before and after chemotherapy. Cancer Chemother Pharmacol. 2014;73:317–24. 24258454 10.1007/s00280-013-2348-x

[43] Ma Y, Ren Y, Dai Z-J, Wu C-J, Ji Y-H, Xu J. IL-6, IL-8 and TNF-α levels correlate with disease stage in breast cancer patients. Adv Clin Exp Med. 2017;26:421–6. 28791816 10.17219/acem/62120

[44] Bickel M. The role of interleukin-8 in inflammation and mechanisms of regulation. J Periodontol. 1993;64:456–60. 8315568

[45] Teijeira A, Garasa S, Ochoa MC, et al. IL8, neutrophils, and NETs in a collusion against cancer immunity and immunotherapy. Clin Cancer Res. 2021;27:2383–93. 33376096 10.1158/1078-0432.CCR-20-1319

[46] Razmkhah M, Jaberipour M, Hosseini A, Safaei A, Khalatbari B, Ghaderi A. Expression profile of IL-8 and growth factors in breast cancer cells and adipose-derived stem cells (ASCs) isolated from breast carcinoma. Cell Immunol. 2010;265:80–5. 20705284 10.1016/j.cellimm.2010.07.006

[47] Schaue D, Micewicz ED, Ratikan JA, Xie MW, Cheng G, McBride WH. Radiation and inflammation. Semin Radiat Oncol. 2015;25:4–10. 25481260 10.1016/j.semradonc.2014.07.007 PMC4378687

[48] van der Most RG, Currie AJ, Robinson BWS, Lake RA. Decoding dangerous death: how cytotoxic chemotherapy invokes inflammation, immunity or nothing at all. Cell Death Differ. 2008;15:13–20. 18007666 10.1038/sj.cdd.4402255

[49] Christensen IB, Ribas L, Buch-Larsen K, Marina D, Larsen S, Schwarz P, Dela F, Gillberg L. Peripheral blood mononuclear cells exhibit increased mitochondrial respiration after adjuvant chemo- and radiotherapy for early breast cancer. Cancer Med. 2022;12(16):16985–96. 10.1002/cam4.6333. 10.1002/cam4.6333 PMC10501284 37439084

[50] Mohamed-Ali V, Goodrick S, Rawesh A, Katz DR, Miles JM, Yudkin JS, Klein S, Coppack SW. Subcutaneous adipose tissue releases interleukin-6, but not tumor necrosis factor-alpha, in vivo. J Clin Endocrinol Metab. 1997;82:4196–200. 9398739 10.1210/jcem.82.12.4450

