# Supplementary material for: Pro-inflammatory cytokines increase temporarily after adjuvant treatment for breast cancer in postmenopausal women: a longitudinal study
Source: Breast Cancer Res. 2024 Oct 16;26:142. doi: 10.1186/s13058-024-01898-3 (PMC11481761; doi:10.1186/s13058-024-01898-3)
Supplement: Supplementary file 1 — Additional file 1. [file 13058_2024_1898_MOESM1_ESM.pdf]

Parameter	EBC patients Total n = 51
Tumor stage	
I	7 (15 %)
II	28 (55 %)
III	16 (31 %)
Histology	
Invasive Ductal Carcinoma	45 (88 %)
Invasive Luminal/Lobular Carcinoma	5 (10 %)
Adenoid cystic carcinoma	1 (2 %)
Surgery type	
Lumpectomy	34 (67 %)
Mastectomy	17 (33 %)
Lymph node involvement	
0	24 (47 %)
1-3	22 (43 %)
4+	4 (8 %)
Missing data	1 (2 %)
ER status	
Positive	42 (82 %)
Negative	9 (18 %)
HER2 status	
Positive	20 (39 %)
Negative	31 (61 %)
Chemotherapy drugs [1]	
Cyclophosphamide	44 (86 %)
Epirubicin	44 (86 %)
Paclitaxel	50 (98 %)
Nab-paclitaxel	2 (4%)
Capecitabine	1 (2 %)
Neoadjuvant chemotherapy (before tumor resection)	12 (24%)
Adjuvant chemotherapy (after tumor resection)	39 (76%)
Radiotherapy	
Yes	41 (80 %)
No	10 (20 %)
Anti-estrogen treatment	
Aromatase inhibitors	40 (78 %)
Tamoxifen	1 (2 %)
None	10 (20%)
Anti-resorptive treatment	
Zoledronic acid	45 (88%)
Denosumab	3 (6%)
None	3 (6%)
Trastuzumab treatment [2]	
Yes	20 (39%)
No	31 (61%)

Supplementary Table 1. **Tumor characteristics and treatment regimens of the 51 EBC patients.** Data are presented as number of patients (percentage of the cohort). EBC: early breast cancer; ER: estrogen receptor; HER2: human epidermal growth factor receptor 2; n: sample size. [1] The majority of patients received paclitaxel (12 weekly infusions) followed by cyclophosphamide and epirubicin (4 infusions every third week). [2] Initiated during the first cycle of cyclophosphamide and epirubicin treatment and administered every third week for 17 cycles in standard doses.

LLOD values	IFN-γ	IL-6	IL-8	IL-10	TNF-α	IL-1β	IL-2	IL-4	IL-12p70	IL-13
**Plate 1**	0.466	0.169	0.048	0.072	0.304	0.033	0.108	0.018	0.058	1.244
**Plate 2**	0.804	0.156	0.049	0.087	0.344	0.054	0.108	0.021	0.076	0.616
**Plate 3**	0.344	0.173	0.054	0.040	0.077	0.036	0.074	0.018	0.063	0.702
**Plate 4**	0.310	0.368	0.216	0.081	0.137	0.042	0.080	0.019	0.060	1.494
**Percentage of samples within detection range**	**100%**	**91%**	**100%**	**94%**	**100%**	**65%**	**75%**	**42%**	**77%**	**22%**
**Percentage of samples with CV >20% on plate 1**	**0%**	**22%**	**0%**	**5%**	**3%**	**47%**	**30%**	**62%**	**66%**	**36%**
**Average CV on plate 1**	**2.8**	**7.0**	**2.9**	**6.4**	**6.3**	**17.6**	**17.0**	**28.7**	**27.8**	**11.6**

Supplementary Table 2. **Quality control of data generated with V-PLEX Pro-inflammatory Panel 1 (human) kit.** A lower level of detection (LLOD) is presented for each cytokine and 96-well plate. The average percentage of samples within the detection range are presented for each cytokine on all plates. On plate 1, 40 samples were run in duplicates. The percentage of samples with a coefficient of variation (CV) above 20% are presented along with the average CV % for all samples on plate 1.

	IL-6	IL-8	IL-10	IFN- γ	TNF-α	hsCRP
**IL-6**	N/A	0.4544 (0.1964 to 0.654) **	0.0301 (-0.255 to 0.311)	0.353 (0.077 to 0.578) *	0.257 (-0.031 to 0.506)	0.697 (0.516 to 0.819)**
**IL-8**	0.454 (0.196 to 0.654) **	N/A	0.283 (-0.0003 to 0.524) *	0.166 (-0.123 to 0.429)	0.381 (0.106 to 0.601) *	0.189 (-0.099 to 0.449)
**IL-10**	0.030 (-0.255 to 0.311)	0.283 (-0.0003 to 0.524) *	N/A	0.082 (-0.206 to 0.357)	0.358 (0.080 to 0.584) *	0.0035 (-0.280 to 0.286)
**IFN-g**	0.353 (0.077 to 0.578) *	0.166 (-0.123 to 0.429)	0.082 (-0.206 to 0.357)	N/A	0.193 (-0.099 to 0.454)	0.260 (-0.025 to 0.506)
**TNF-a**	0.257 (-0.031 to 0.506)	0.3808 (0.106 to 0.601) *	0.358 (0.080 to 0.584) *	0.193 (-0.099 to 0.454)	N/A	0.259 (-0.029 to 0.507)
**hsCRP**	0.698 (0.516 to 0.819) **	0.189 (-0.099 to 0.449)	0.003 (-0.280 to 0.286)	0.260 (-0.025 to 0.506)	0.259 (-0.029 to 0.507)	N/A

Supplementary Table 3. Correlations between plasma cytokine and hsCRP levels in postmenopausal EBC patients before initiation of adjuvant therapy. Results are presented as Spearman correlation coefficients (r) with 95% confidence intervals. * p<0.05. ** p<0.005.

	IL-6	IL-8	IL-10	IFN-γ	TNF-α	hsCRP
Fasting insulin (pmol/L)	0.316 (0.019 to 0.561) *	0.059 (-0.244 to 0.351)	-0.072 (-0.362 to 0.232)	-0.057 (-0.349 to 0.246)	0.018 (-0.285 to 0.318)	0.396 (0.111 to 0.621) *
HOMA-IR	0.313 (0.017 to 0.560) *	0.051 (-0.251 to 0.344)	-0.041 (-0.335 to 0.261)	-0.044 (-0.338 to 0.258)	-0.021 (-0.321 to 0.283)	0.368 (0.079 to 0.601) *
HDL cholesterol (mmol/L)	-0.350 (-0.576 to -0.074) *	-0.219 (-0.473 to 0.069)	-0.163 (-0.427to 0.126)	-0.204 (-0.461 to 0.084)	-0.477 (-0.671 to -0.221) **	-0.180 (-0.441 to 0.108)
Triglyceride (mmol/L)	0.521 (0.270 to 0.706) **	0.221 (-0.076 to 0.482)	-0.075 (-0.359 to 0.222)	0.327 (0.039 to 0.565) *	0.129 (-0.173 to 0.409)	0.307 (0.017 to 0.550) *
Hemoglobin (mmol/L)	0.378 (0.074 to 0.617) *	0.375 (0.071 to 0.615)*	0.053 (-0.264 to 0.360)	0.168 (-0.152 to 0.457)	0.299 (-0.018 to 0.562)	0.287 (-0.027 to 0.550)
Leucocytes (10^9/L)	0.561 (0.302 to 0.743) **	0.066 (-0.251 to 0.371)	0.043 (-0.273 to 0.351)	0.266 (-0.051 to 0.534)	0.121 (-0.203 to 0.421)	0.559 (0.299 to 0.742) **

Supplementary Table 4. Correlations between cytokine and hsCRP levels in postmenopausal EBC patients before initiation of adjuvant therapy and the clinical characteristics that significantly change with adjuvant therapy. Results are presented as Spearman correlation coefficients (r) with 95% confidence intervals. LDL: Low density lipoprotein. HDL: High density lipoprotein. HOMA-IR: Homeostatic model assessment for insulin resistance. * p<0.05. ** p<0.005.

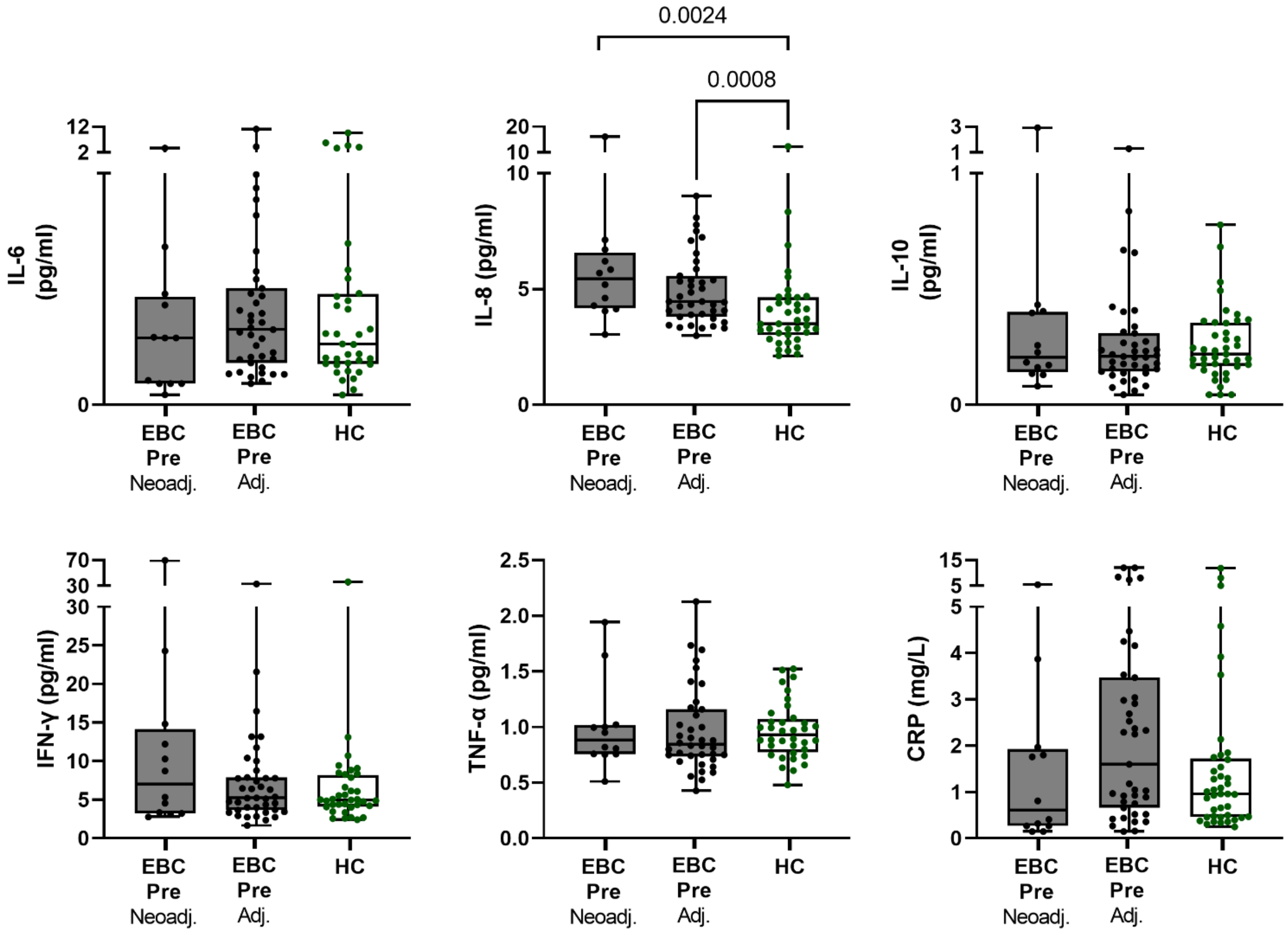

Supplementary Figure 1. Cytokine and hsCRP levels in EBC patients who had a tumor present (Neoadj., n=12) or already had tumor resection (Ajd., n=39) and in age- and BMI-matched healthy controls (n=41). Data are presented by boxplots indicating medians, interquartile range (25th to 75th percentile) and minimum to maximum (whiskers). Differences between groups were analyzed with Mann-Whitney tests. Ajd., adjuvant chemotherapy (no tumor present at blood sampling). EBC, early breast cancer. HC, healthy controls. Neoadj., neoadjuvant chemotherapy (tumor present at blood sampling). Pre, before chemotherapy.

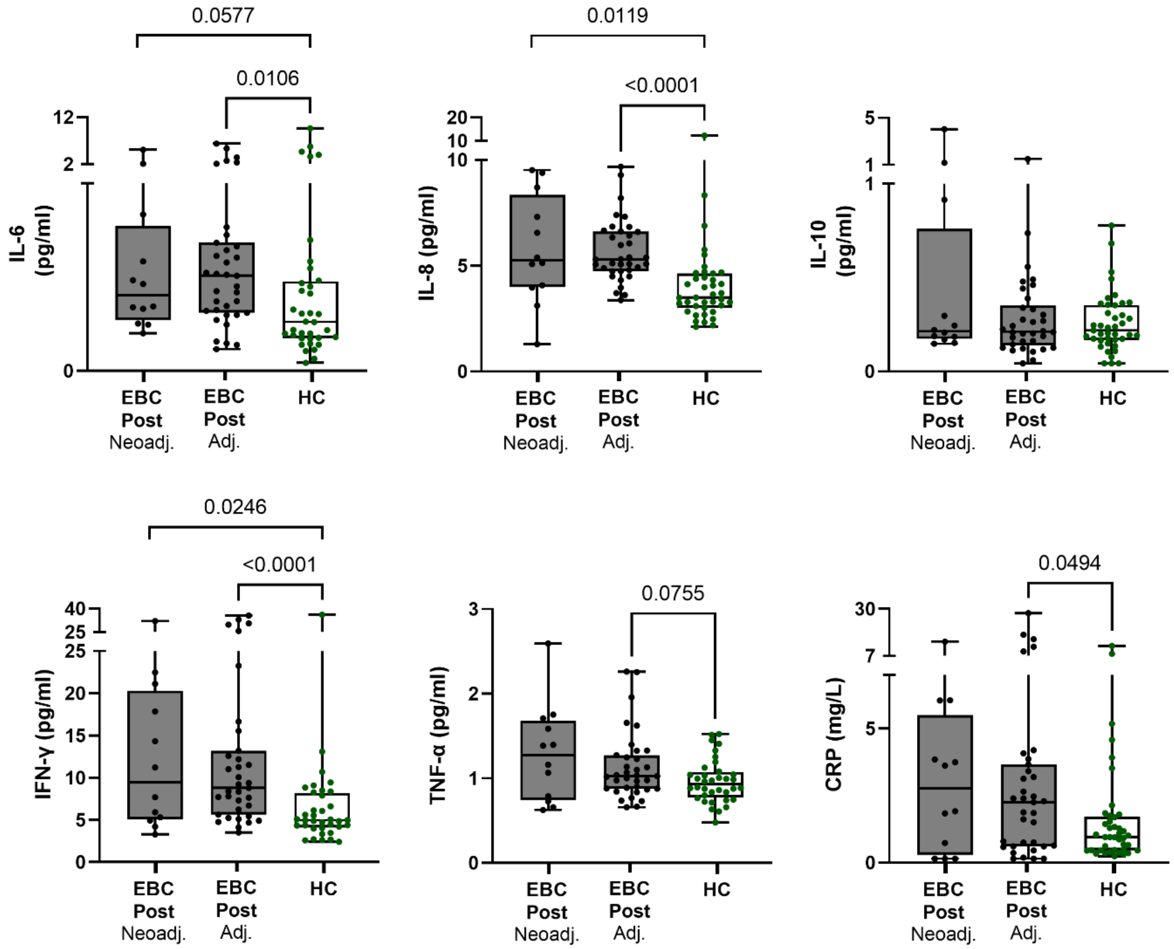

Supplementary Figure 2. Cytokine and hsCRP levels in EBC patients after treatment with neoadjuvant chemotherapy (Neoadj. n=12) or adjuvant chemotherapy (Ajd., n=39) and in age- and BMI-matched healthy controls (n=41). Data are presented by boxplots indicating medians, interquartile range (25th to 75th percentile) and minimum to maximum (whiskers). Differences between groups were analyzed with Mann-Whitney tests. Ajd., adjuvant chemotherapy. EBC, early breast cancer. HC, healthy controls. Neoadj., neoadjuvant chemotherapy. Post, shortly after chemotherapy.

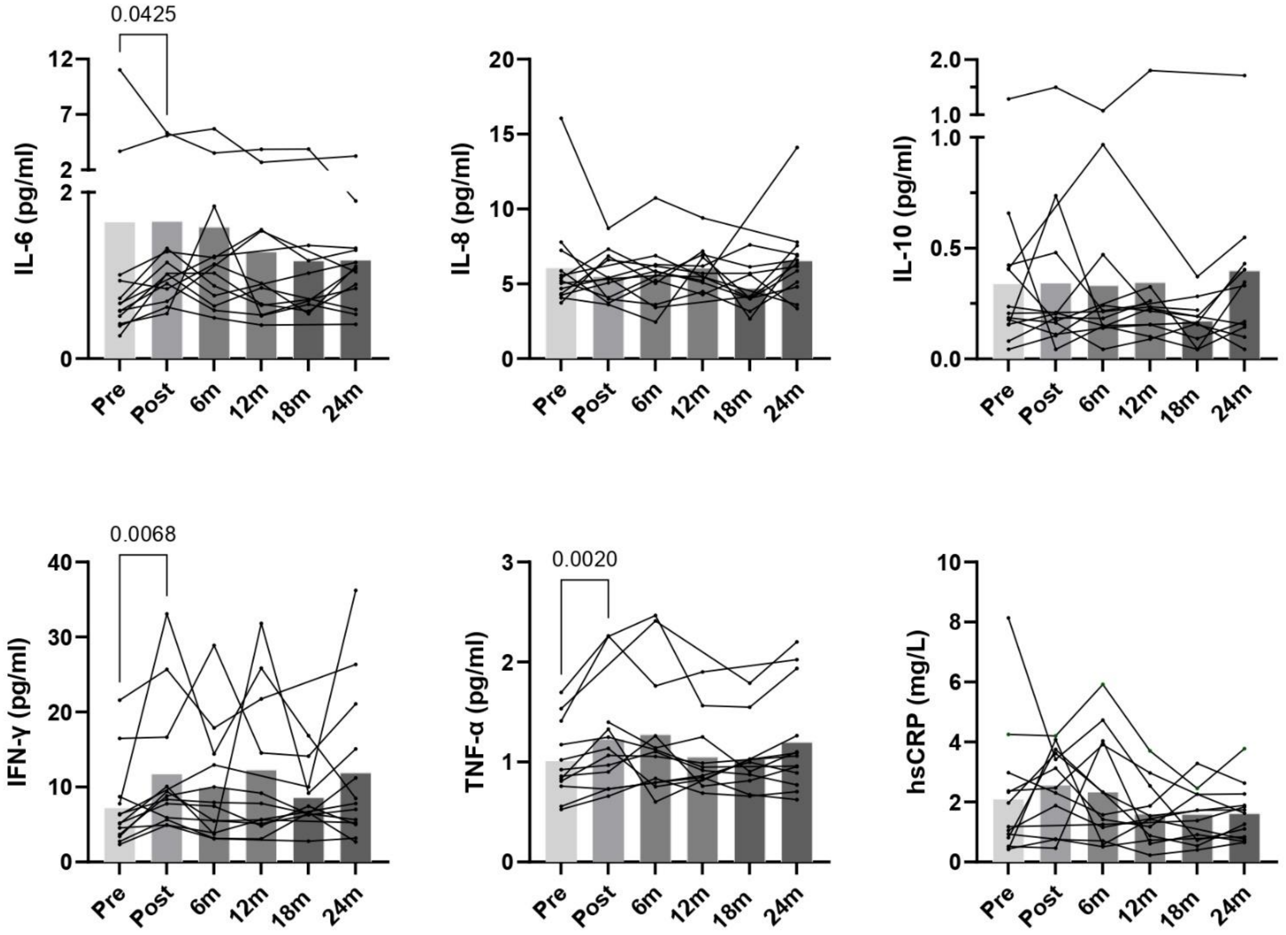

Supplementary Figure 3. Cytokine and hsCRP fluctuations for the 13 EBC patients who were examined during 24 months after chemotherapy completion. P-values are based on Wilcoxon tests (Pre vs Post). hsCRP, high-sensitivity C-reactive protein. Pre, before chemotherapy. Post, after chemotherapy.

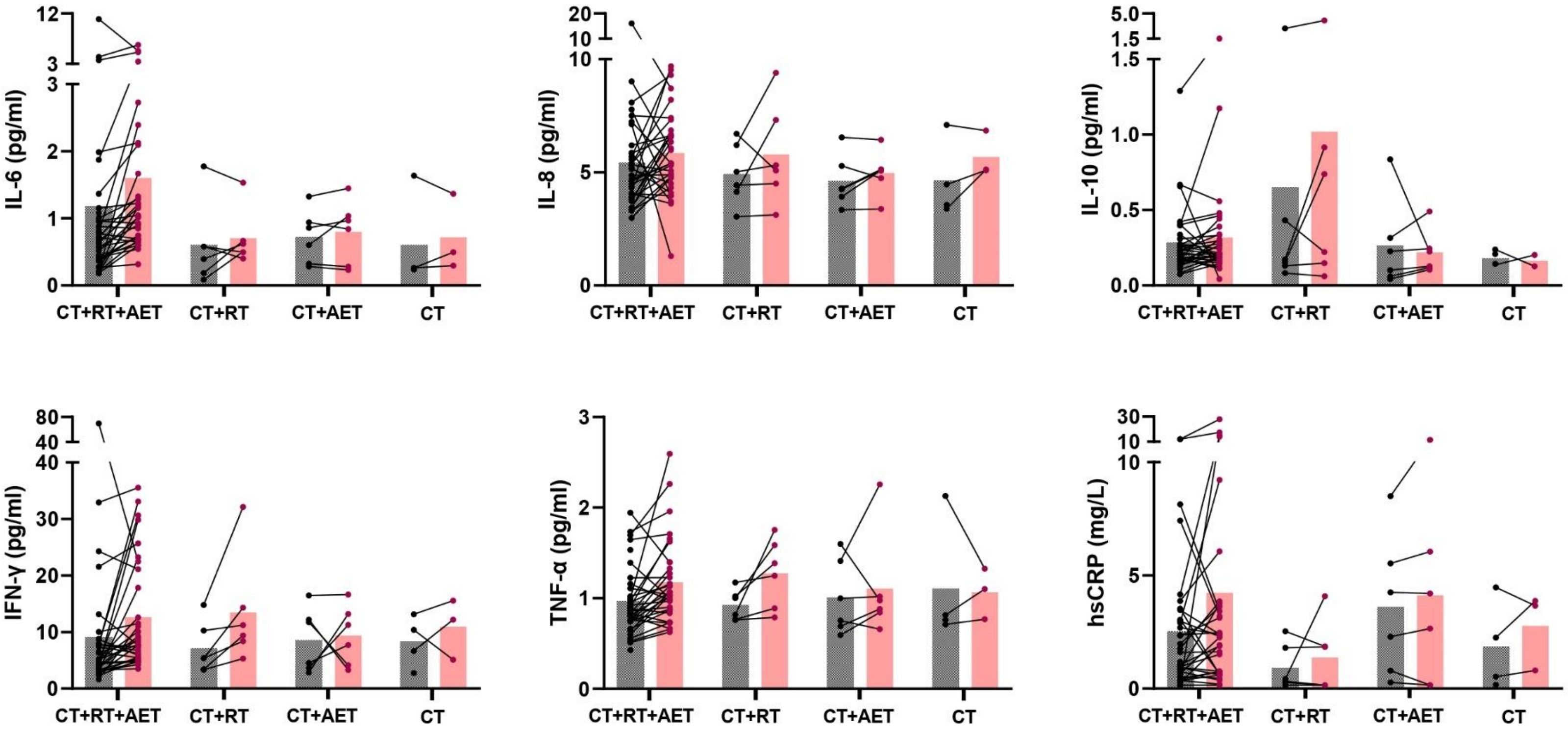

Supplementary Figure 4. Cytokine and hsCRP levels before versus after chemotherapy with or without radio- and/or anti-estrogen therapy. EBC patients were investigated before (Pre, grey bars) and after (Post, pink bar) chemo-, radio- and anti-estrogen therapy (CT+RT+AET, n=35), chemo- and radiotherapy (CT+RT, n=6), chemo- and anti-estrogen therapy (CT+AET, n=6) or chemotherapy only (CT, n=4). Pre and Post values for each patient are indicated by a line. Statistical analyses have not been performed due to a low number of patients in most of the groups. AET, anti-estrogen therapy. hsCRP, high-sensitivity C-reactive protein. CT, radiotherapy. EBC, early breast cancer. Pre, before chemotherapy. Post, after chemotherapy. RT, radiotherapy.
